# Meaningful score changes for SF-36v2, FACIT-fatigue, and RASIQ in rheumatoid arthritis

**DOI:** 10.1186/s41687-024-00685-0

**Published:** 2024-01-22

**Authors:** Regina Rendas-Baum, Xiaochen Lin, Mark Kosinski, Jakob Bue Bjorner, Marguerite G. Bracher, Wen-Hung Chen

**Affiliations:** 1 QualityMetric Incorporated, LLC, Johnson, RI USA; 2 GSK, Global Value Evidence & Outcomes, Stevenage, Hertfordshire UK; 3 GSK, Global Value Evidence & Outcomes, 1250 South Collegeville Road, Building 4, 4th floor, 19426 Collegeville, PA USA; 4 Biogen, Cambridge, MA USA

**Keywords:** Rheumatoid arthritis, Within-patient meaningful improvement, Interpretation thresholds, SF-36v2, RASIQ, FACIT-Fatigue

## Abstract

**Background:**

Interpretation thresholds for patient-reported outcome (PRO) scores are of crucial importance, particularly when interpreting treatment benefit. This study was designed to determine the within-patient meaningful improvement (WPMI) thresholds for the Short-Form 36 Health Survey version 2 (SF-36v2), the Functional Assessment of Chronic Illness Therapy-Fatigue (FACIT-Fatigue), and the novel Rheumatoid Arthritis Symptoms and Impact Questionnaire (RASIQ) among patients with rheumatoid arthritis (RA).

**Methods:**

In this *post-hoc* analysis, anchor-based and supportive distribution-based methods were used to derive WPMI based on blinded data from all treatment arms in two Phase 2 RA trials with otilimab. Patient’s Global Assessment of Disease Activity (PtGA) was the general anchor for all SF-36v2 scales. SF-36 Patient’s Global Impression of Status (PGIS), PtGA, and VT03 (an SF-36v2 item) were used as anchors for FACIT-Fatigue. SF-36 PGIS, PtGA, and Patient’s Assessment of Arthritis Pain (PAIN) were anchors for RASIQ. Mean change was calculated for the anchor category associated with minimal meaningful improvement from baseline to Week 24 for SF-36v2 and FACIT-Fatigue, and to Week 12 for RASIQ. Sensitivity and specificity were used to evaluate the accuracy of estimated WPMI values.

**Results:**

For the SF-36v2 physical functioning, role physical, bodily pain, general health, vitality, social functioning, role emotional, and mental health domains, anchor-based estimates of WPMI based on 0–100 scores were 24.5, 24.5, 25.4, 13.6, 21.5, 20.5, 16.9, and 14.3, respectively. Anchor-based WPMI estimates were 9.7 for the Physical Component Summary score and 7.6 for the Mental Component Summary score (using norm-based T-score metric). For FACIT-Fatigue (range 0–52), WPMI estimates ranged from 9.7 to 11.3 points. For RASIQ (range 0–100), anchor-based WPMI was determined as a change between -32.7 and -21.7 points for the Joint Pain scale, -26.7 to -23.7 for the Joint Stiffness scale, and -21.1 to -17.4 for the Impact scale.

**Conclusions:**

This study derived WPMI thresholds for SF-36v2, FACIT-Fatigue, and RASIQ among patients with RA, using multiple anchors. Derivation of WPMI thresholds for these PRO instruments will enable their broader use in evaluating and interpreting treatment benefit in future RA studies.

**Supplementary Information:**

The online version contains supplementary material available at 10.1186/s41687-024-00685-0.

## Abstract (plain language summary)

When assessing medical treatments in clinical trials, it is important to understand whether the treatment improves symptoms or impacts of a disease to an extent which is meaningful for patients. Patients are often asked to complete questionnaires about their symptoms throughout clinical trials to measure if and how symptoms change. Questionnaire responses are used to calculate a score that is compared before and after treatment. This study was designed to investigate how much scores in three questionnaires (SF-36v2, FACIT-Fatigue, and RASIQ) changed for patients with rheumatoid arthritis who reported experiencing meaningful symptom improvement based on data from two clinical trials. As the RASIQ is a new questionnaire that was designed specifically for rheumatoid arthritis, this research is particularly important for interpretation of RASIQ results.

**Supplementary Information:**

The online version contains supplementary material available at 10.1186/s41687-024-00685-0.

## Background

Thresholds of meaningful score change for patient-reported outcomes (PROs) are of crucial importance, particularly when assessing and interpreting treatment benefit. Within-patient meaningful improvement (WPMI) represents the smallest difference in an outcome measure which is considered by patients to be beneficial [1, 2]. As recommended by the United States Food and Drug Administration, appropriate thresholds that indicate clinically meaningful within-patient change should be established *a priori* via anchor-based methods, using anchors such as the Patient Global Impression of Severity [3]. These thresholds for WPMI can subsequently be used to interpret clinical trial data.

The Short-Form 36 Health Survey version 2 (SF-36v2) and Functional Assessment of Chronic Illness Therapy-Fatigue (FACIT-Fatigue) are both PRO instruments used to quantify key concepts important to patients with various diseases, including rheumatoid arthritis (RA) [4]. Although both are well-established instruments, this study aims to contribute to the ability to interpret results obtained from these instruments by estimating thresholds for WPMI. More recently, the Rheumatoid Arthritis Symptoms and Impact Questionnaire (RASIQ) was developed to specifically evaluate the symptoms of RA and their impact on patients [5]. Establishing interpretation thresholds of the score change for both new and established PRO instruments furthers understanding of results obtained from these PROs.

This *post-hoc* analysis used data from the Phase 2 BAROQUE (NCT02504671) [6] and RENAISSANCE (NCT02799472) [7] otilimab trials to determine the WPMI thresholds for SF-36v2, FACIT-Fatigue, and RASIQ among patients with RA. This study extends prior work by comparing previously established interpretation thresholds for SF-36v2 and FACIT-Fatigue [8–10] to those obtained using data from the otilimab trials, and establishing WPMI thresholds for RASIQ.

## Methods

WPMI thresholds for SF-36v2, FACIT-Fatigue, and RASIQ were established using anchor-based methods, with supportive distribution-based methods and measures of accuracy (sensitivity and specificity) used to further triangulate across the estimates obtained from different anchors. Cumulative distribution function (CDF) plots were also generated to illustrate how well anchor-based change categories were separated across the entire range of RASIQ scale change scores.

### Survey content and scoring

The SF-36v2 is a 36-item, self-report survey of functional health and well-being that is scored as two component summary scores (physical and mental health) and as eight domain scores; physical functioning (PF), role limitations due to physical health (RP), bodily pain (BP), general health perceptions (GH), vitality (VT), social functioning (SF), role limitations due to emotional problems (RE), and mental health (MH) [11].

For the eight domain scores, results are presented using a score range from 0 (worst possible health) to 100 (best possible health). Additional File 1 reports results for norm-based scores (NBS), which standardize scale and component scores using the means and standard deviations (SD) from a US general population normative sample [11]. The Physical Component Summary (PCS) and Mental Component Summary (MCS) scores are always based on NBS, using a mean of 50 and a SD of 10 in the US adult general population, with higher scores indicating better health.

The 13-item FACIT-Fatigue questionnaire assesses self-reported fatigue and its impact upon daily activities and function over the past 7 days; item responses are added with equal weight to obtain the total score which ranges from 0 (most fatigue) to 52 (least fatigue) [12].

RASIQ is a novel measure comprised of 16 items across three domains (Joint Pain [JP], Joint Stiffness [JS], and Impact [IM]). Scores from each item are summed and transformed to a metric ranging from 0 (least pain/stiffness/impact) to 100 (most pain/stiffness/impact) [5].

### Data sources

BAROQUE [6] was a randomized, Phase 2b, dose-adaptive, multi-center, double-blind, placebo-controlled trial which assessed the efficacy of the anti-granulocyte-macrophage colony-stimulating factor monoclonal antibody, otilimab, in patients with active, moderate-to-severe RA despite treatment with methotrexate. RENAISSANCE [7] was a Phase 2a, multi-center, double-blind, placebo-controlled trial which evaluated change from baseline in various exploratory biomarkers among patients with RA treated with otilimab. While both trials included the RASIQ, the SF-36v2 and FACIT-Fatigue were only used in the BAROQUE trial, and completed at baseline, Weeks 4, 12, 24, 36, and 52, and follow-up. The RASIQ was completed at screening, baseline, Weeks 1, 6, and 12, and follow-up in the RENAISSANCE study, and at Weeks 1, 12, 24, 36, and 52, and follow-up in the BAROQUE trial.

Data from baseline to Week 24 in the BAROQUE trial were used in the SF-36v2 and FACIT-Fatigue analyses. Pooled data from baseline to Week 12 in the BAROQUE and RENAISSANCE trials were used in the analyses of RASIQ.

### Anchor items

The general anchor for all SF-36v2 scales was the Patient’s Global Assessment of Disease Activity (PtGA), with scores ranging from 0 (very well) to 100 (very poor). In addition, Patient’s Assessment of Arthritis Pain (PAIN; scores range from 0 [no pain] to 100 [most severe pain]) was used as an anchor for the BP scale. One item from the FACIT-Fatigue questionnaire, AN5 (*I have energy*; *Not at all / A little bit / Somewhat / Quite a bit / Very much*), was used as an anchor for the VT scale.

The PtGA item and two items from the SF-36v2 were used as anchors for FACIT-Fatigue. The first SF-36v2 item assessed the Patient’s Global Impression of Status (PGIS; *In general, would you say your health is: Excellent / Very good / Good / Fair / Poor*), and the second item focused on fatigue (*How much of the time during the past 4 weeks did you feel worn out? Not at all / A little bit / Somewhat / Quite a bit / Very much*).

WPMI analyses of RASIQ were based on the SF-36 PGIS, PtGA, PAIN, and additional items on pain and overall impact. The SF-36 PGIS and PtGA were used as anchors for all RASIQ scales. The PAIN and one SF-36v2 item focused on pain (*How much bodily pain have you had during the past 4 weeks? None / Very mild / Mild / Moderate / Severe / Very severe*) were used as additional anchors for the JP scale, and two FACIT-Fatigue items (*I feel tired* and *I feel listless [washed out]: response scale for both; Not at all / A little bit / Somewhat / Quite a bit / Very much*) were used as additional anchors for the IM scale. Full details of the anchors used are shown in Table 1.

**Table 1:** Anchors used for SF-36v2, FACIT-Fatigue, and RASIQ^a^

	Anchor							
**PRO scales**	**PGIS** ^b^	**PtGA** ^c^	**PAIN** ^d^	**BP01** ^e^	**AN5** ^f^	**AN2** ^g^	**AN1** ^h^	**VT03** ^i^
**SF-36v2 domains**								
Physical functioning		-0.44						
Role physical		-0.43						
Bodily pain		-0.48	-0.55					
General health		-0.29						
Vitality		-0.42			-0.38			
Social functioning		-0.30						
Role emotional		-0.22						
Mental health		-0.24						
Physical Component Summary		-0.47						
Mental Component Summary		-0.22						
**RASIQ scales**								
Joint Pain	-0.44	0.69	0.75	-0.56				
Joint Stiffness	-0.46	0.51						
Impact	-0.57	0.59				-0.60	-0.49	
**FACIT-Fatigue**								
Fatigue	0.48	-0.44						0.58

^a^Values in table cells represent Spearman correlation coefficients between change in PRO scale and change in anchor. Correlations for SF-36v2 and FACIT-Fatigue are based on data from the BAROQUE clinical trial. Correlations for RASIQ are based on pooled data from both the BAROQUE and RENAISSANCE clinical trials

^b^In general, would you say your health is… [Excellent/Very good/Good/Fair/Poor]

^c^Considering all the ways your arthritis has affected you, how active do you feel your arthritis is… [numeric scale from 0 (very well) to 100 (very poor)]

^d^How much pain are you currently having because of your rheumatoid arthritis? [numeric scale from 0 (no pain) to 100 (most severe pain)]

^e^How much bodily pain have you had during the past 4 weeks? [None/Very mild/Mild/Moderate/Severe/Very severe]

^f^I have energy [Not at all/A little bit/Somewhat/Quite a bit/Very much]

^g^I feel tired [Not at all/A little bit/Somewhat/Quite a bit/Very much]

^h^I feel listless (washed out) [Not at all/A little bit/Somewhat/Quite a bit/Very much]

^i^How much of the time during the past 4 weeks did you feel worn out? [None of the time/A little of the time/Some of the time/Most of the time/All of the time]

AN: anchor; BP: bodily pain; FACIT-Fatigue: Functional Assessment of Chronic Illness Therapy-Fatigue; NBS: norm-based scores; PAIN: Patient’s Assessment of Arthritis Pain; PGIS: Patient’s Global Impression of Status; PtGA: Patient’s Global Assessment of Disease Activity; PRO: patient-reported outcomes; RASIQ: Rheumatoid Arthritis Symptoms and Impact Questionnaire; SF-36v2: Short-Form 36 Health Survey version 2; VT: vitality

For categorical anchors, a one-point (or one-category) improvement was deemed to be associated with the smallest meaningful change indicating improvement. The categorizations of change groups for anchors that used a continuous metric were based on results from studies that established thresholds for within-person change for the same measure and among a sample of patients with RA [13]. For PtGA, a value of -18 was used, and for PAIN, a value of -20 was used.

### Statistical analysis

The association between change in each PRO score and the proposed anchors was evaluated using the Spearman correlation coefficient with a recommended value of at least 0.30 indicating adequacy of the anchor [14, 15]. WPMI was estimated as the mean score change from baseline to Week 12 or 24 in the group associated with the smallest meaningful improvement in each corresponding anchor. Effect sizes were calculated using standardized response mean (SRM) to better compare the magnitude of the mean change scores, using:

$$ SRM=\frac{{\stackrel{-}{X}}_{change}}{{SD}_{change}} $$

where the numerator consists of the mean of the change scores and the denominator is the SD of the same change score.

The reliable change index (RCI) was used to identify change that can be considered beyond measurement error [16]. First, the standard error of the measurement (SEM) was calculated using:

$$ SEM= {SD}_{baseline}\text{*}\sqrt{1-reliability }$$

Reliability was estimated using Cronbach’s alpha [17], a measure based on inter-item correlations. As a sensitivity analysis, reliability was also estimated using the omega coefficient [18] and the greatest lower bound [19]. These analyses gave very similar results. Next, the RCI was calculated using:

$$ RCI= \sqrt{2 } \times SEM \times 1.282$$

In the equation above, 1.282 is taken from the standardized normal distribution; it represents the half-width of the 80% confidence interval, which is a reasonable criterion for individual respondents proviiding an appropriate balance between the risks of falsely identifying change and overlooking true change [11]. Half of a standard deviation (based on baseline scores) is also reported for completeness, as this has been advocated by researchers in the field [20].

Sensitivity and specificity were used as measures of accuracy to characterize and compare the various anchor-based estimates. Sensitivity indicates the likelihood of correctly identifying a truly improved individual, while specificity indicates the likelihood that an individual that has not improved is correctly classified as such. For the current analyses, the anchor was used as the gold-standard, while the PRO measure was used as the classification or ‘test’ variable.

The CDF plots of change scores were used to better understand the separation between anchor-based change groups across the entire range of observed RASIQ change scores. CDF plots were obtained for each RASIQ scale using the respective anchors, focusing on the anchor category where the patients are defined by the anchor measure as having experienced meaningful change. A consistent separation across the score range between the curve for this category of change and those of adjacent groups indicates support for the anchor.

## Results

### Estimated WPMIs of SF-36v2

The correlation between the SF-36v2 PF, RP, BP, VT, SF, and PCS change scores and change in PtGA ranged between -0.30 and -0.48 (absolute value), as shown in Table 1. For the four remaining SF-36v2 scales (GH, RE, MH, and MCS), correlations ranged between -0.22 and -0.29, indicating that the PtGA is not an empirically adequate anchor for these scales.

Anchor-based WPMI values for the eight SF-36v2 0–100 domain scores ranged between 13.6 for the GH scale (with an SRM of 0.87) and 26.6 for the BP scale (with an SRM of 1.73) (Table 2). PCS and MCS had WPMI estimates based on NBS of 9.7 and 7.6, and SRM of 1.47 and 0.70, respectively. The accuracy measures of these threshold values for identifying meaningful improvement indicated that for most scales, the thresholds have better sensitivity (0.66 to 0.87) than specificity (0.43 to 0.58).

**Table 2:** Anchor- and distribution-based estimates for the SF-36v2, FACIT-fatigue, and RASIQ

	**Anchor-based** **WPMI** **estimates**							Distribution-based estimates		Reliability^i^
	Anchor	**N**	Mean	95% CI	SRM	Sensitivity	Specificity	0.5 SD	RCI(80)	
**SF-36v2 scale (0–100 scoring)**										
PF	PtGA^a^	120	24.5	21.1, 28.0	1.27	0.78	0.56	9.8	12.7	0.87
RP	PtGA^a^	120	24.5	20.6, 28.5	1.37	0.77	0.58	9.1	10.7	0.89
BP	PtGA^a^	120	25.4	21.9, 28.9	1.65	0.80	0.43	7.6	11.1	0.84
	PAIN^b^	125	26.6	23.3, 29.9	1.73	0.87	0.47			
GH	PtGA^a^	120	13.6	10.8, 16.4	0.87	0.74	0.45	8.2	16.7	0.68
VT	PtGA^a^	120	21.5	18.2, 24.8	1.30	0.75	0.50	8.3	13.1	0.81
	AN5^c^	60	23.3	18.7, 28.0	1.41	0.71	0.55			
SF	PtGA^a^	120	20.5	16.3, 24.7	0.98	0.66	0.51	10.5	19.7	0.74
RE	PtGA^a^	120	16.9	12.9, 21.0	0.70	0.68	0.45	12.4	12.1	0.93
MH	PtGA^a^	120	14.3	11.3, 17.3	0.75	0.69	0.53	9.5	13.3	0.85
**SF-36v2 summary (NBS scoring)**										
PCS	PtGA^a^	120	9.7	8.3, 11.1	1.47	0.83	0.47	3.4	4.1	0.89
MCS	PtGA^a^	120	7.6	5.8, 9.4	0.70	0.66	0.50	5.4	6.0	0.91
**FACIT-Fatigue (0–52 scoring)**										
Fatigue	SF-36 PGIS^d^	120	11.3	9.7, 12.9	1.15	0.81	0.52			0.92
	PtGA^a^	80	9.7	8.1, 11.4	0.99	0.75	0.58	4.9	4.9	
	VT03^e^	74	11.1	9.4, 12.9	1.13	0.85	0.55			
**RASIQ (0–100 scoring)**										
Joint pain	SF-36 PGIS^d^	66	-24.0	-28.4, -19.7	-1.34	0.73	0.56	9.1	-6.8	0.96
	PtGA^a^	104	-31.0	-34.6, -27.5	-1.70	0.94	0.49			
	PAIN^b^	97	-32.7	-36.2, -29.3	-1.80	0.94	0.51			
	BP01^f^	71	-21.7	-25.4, -18.0	-1.21	0.84	0.58			
Joint stiffness	PGIS^d^	66	-23.3	-27.4, -19.1	-1.31	0.73	0.62	9.1	-13.1	0.84
	PtGA^a^	104	-26.1	-29.8, -22.3	-1.44	0.83	0.47			
Impact	SF-36 PGIS^d^	66	-21.0	-24.5, -17.4	-1.35	0.89	0.48	7.8	-12.8	0.80
	PtGA^a^	104	-21.1	-24.3, -17.9	-1.35	0.93	0.44			
	AN2^g^	70	-17.4	-20.7, -14.0	-1.12	0.85	0.57			
	AN1^h^	61	-17.8	-21.7, -13.8	-1.14	0.79	0.58			

^a^‘Considering all the ways your arthritis has affected you, how active do you feel your arthritis is…’. Improvement: a decline of 18 points or more

^b^‘How much pain are you currently having because of your rheumatoid arthritis?’ Improvement: a decline of 20 points or more

^c^‘I have energy’. Improvement: an increase of 1 point on a 5-point Likert scale

^d‘^In general, would you say your health is…’. Improvement: an increase of 1 point on a 5-point Likert scale

^e^‘How much of the time during the past 4 weeks did you feel worn out?’ Improvement: an increase of 1 point on a 5-point Likert scale

^f^‘How much bodily pain have you had during the past 4 weeks?’ Improvement: an increase of 1 point on a 5-point Likert scale

^g^‘I feel tired’. Improvement: an increase of 1 point on a 5-point Likert scale

^h^‘I feel listless (washed out)’. Improvement: an increase of 1 point on a 5-point Likert scale

^i^Cronbach’s alpha, except for SF-36v2 summary scores (PCS, MCS), where reliability is estimated using a method that accounts for the reliability of each score as well as covariances among the scores

AN: anchor; BP, bodily pain; CI: confidence interval; FACIT-Fatigue: Functional Assessment of Chronic Illness Therapy-Fatigue; GH: general health perceptions; MCS: Mental Component Summary; MH: role limitations due to emotional problems; PAIN: Patient’s Assessment of Arthritis Pain; PCS: Physical Component Summary; PF: physical functioning; PGIS: Patient’s Global Impression of Status; PtGA: Patient’s Global Assessment of Disease Activity; RASIQ: Rheumatoid Arthritis Symptoms and Impact Questionnaire; RCI(80): reliable change index based on 80% confidence interval; RE: role limitations due to emotional problems; RP: role limitations due to physical health; SD: standard deviation; SF: social functioning; SF-36v2: Short-Form 36 Health Survey version 2; SRM: standardized response mean; VT: vitality; WPMI: within-patient meaningful improvement

RCI-based estimates were 12.7 for PF, 10.7 for RP, 11.1 for BP, 16.7 for GH, 13.1 for VT, 19.7 for SF, 12.1 for RE, 13.3 for MH, 4.1 for PCS, and 6.0 for MCS (Table 2). Estimates based on 0.5 SD were 9.8, 9.1, 7.6, 8.2, 8.3, 10.5, 12.4, 9.5, 3.4, and 5.4, respectively.

WPMI estimates based on mean change were generally similar, although slightly smaller in some cases (e.g., for the MH scale), to those provided by the cut point associated with the best balance between sensitivity and specificity (Additional File 2, Supplementary Table S1). CDF curves generally mirrored correlation values, with PtGA-based curves being less separated for GH, SF, and RE domains (Additional File 2, Supplementary Figure S1); similarly, PtGA-based CDF curves for PCS were more clearly separated when compared to MCS (Additional File 2, Supplementary Figure S2).

### Estimated WPMIs of FACIT-fatigue

Anchor-based WPMI estimates ranged from 9.7 to 11.3 (SRM 0.99–1.15; Table 2). RCI generated a value of 4.9. The cut point associated with the best sensitivity/specificity balance was slightly smaller than the values obtained with mean change analyses (Additional File 2, Supplementary Table S2). A clear separation between all CDF curves was observed (Additional File 2, Supplementary Figure S3).

### Estimated WPMIs of RASIQ

#### Joint pain scale

Analysis of mean change scores for a one-point improvement in the SF-36 PGIS indicated that a meaningful improvement in the RASIQ’s JP scale was equal to a 24.0-point reduction in score (Table 2). The estimate for the BP anchor (BP01) was -21.7 while the two anchors that are based on a binary categorization of a continuous scale (PAIN and PtGA) provided higher WPMI estimates (-32.7 and -31.0, respectively).

RCI generated a value of -6.8. A clear separation between the CDF curves was observed (Additional File 2, Supplementary Figure S4).

For the values found in the anchor-based analyses (Table 2), sensitivity (range: 0.73–0.94) was higher than specificity (range: 0.49–0.58). For example, with SF-36 PGIS as the anchor, at a threshold of -24.0, the sensitivity was 0.73 while the specificity was 0.56, indicating better performance at correctly classifying patients who have improved than those who have not improved.

#### Joint stiffness scale

Analysis of mean change scores based on an improvement of one point or better in SF-36 PGIS indicated that a meaningful improvement in the RASIQ JS scale was equal to a 23.3-point reduction in score (Table 2). When using PtGA as the anchor, the estimate was approximately 3 points higher (-26.1) in absolute value. Estimates based on RCI (-13.1) were approximately half of those obtained under the mean change score analysis, with the estimate based on 0.5 SD equal to -9.1.

A clear separation between the CDF curves was observed (Additional File 2, Supplementary Figure S5).

#### Impact scale

Analysis of mean change scores based on an improvement of at least one point in SF-36 PGIS indicated that a meaningful improvement in the RASIQ’s Impact scale translated to a 21.0-point reduction in score, which was nearly identical to the estimate obtained under the PtGA anchor (Table 2). The remaining two anchors, AN2 (*I feel tired*) and AN1 (*I feel listless*), resulted in estimates that were slightly smaller (in absolute value) at -17.4 and − 17.8, respectively. The 0.5 SD and RCI criteria resulted in values of -7.8 and -12.8, respectively.

The CDF plots indicate that the curves obtained for each change group were generally separated, except for the plot corresponding to the AN1 anchor (Additional File 2, Supplementary Figure S6).

At a threshold of -21.0, the sensitivity and specificity were 0.89/0.48 when SF-36 PGIS was the anchor; the estimate of -21.1 associated with the PtGA anchor resulted in values of sensitivity and specificity equal to 0.93 and 0.44, respectively. For the smaller WPMI estimates based on the two FACIT-Fatigue items– AN2 and AN1– sensitivity values were slightly lower (0.85/0.79) and specificity slightly higher (0.57/0.58).

## Discussion

Understanding the thresholds for within-patient meaningful change scores for PRO instruments is important for assessing and interpreting benefits of a treatment. In this study, we sought to determine the WPMI thresholds for SF-36v2, FACIT-Fatigue, and RASIQ among patients with RA. As the RASIQ is a new questionnaire that was designed specifically for RA, this research will allow increased use of the measure in the future.

For SF-36v2 NBS scores, most of the WPMI estimates obtained in the current study (using mean change score in the anchor category) were similar or up to 2 times greater than those recommended by the developers, which were derived from the US general population (average number of chronic conditions reported was 2.6 [SD = 2.5]) [11]. It should be noted that the thresholds for within-individual change recommended by the developers were based on SEM around the change score (similar to RCI) rather than confidence intervals for observed change based on patient-rated anchors. WPMI estimates for SF-36v2 items based on 0–100 scores were substantially greater (by a magnitude of 3 to 5 times) than those that have been applied to RA trial data, which identify meaningful within-patient change using a change score of 5 points for the eight SF-36v2 scales [21, 22]. For FACIT-Fatigue, the WPMI estimates ranged between 9.7 and 11.3; again, these estimates are higher than those used in previous studies with patients with RA [22]. A couple of factors should be noted as likely contributors to overestimation of WPMI. Firstly, the operationalization of PtGA and PAIN (i.e., their dichotomization) did not distinguish between a large and a small improvement in health status; however, the analysis of mean change assumes a category of small but meaningful change. In addition, simulation studies have shown that mean change analyses often overestimate the threshold for meaningful change [23].

Factors beyond methodological aspects of the analyses should be noted as potential drivers of the differences between current and previous results. Earlier studies have used different methods/anchors, whereas the anchors used in this study were specific for patients with moderate/severe RA. Patient demographic and clinical characteristics can also influence WPMI estimates. In addition, the commonly used 5-point thresholds for 0–100 scores of the eight domains of the SF-36v2 and 2.5-point thresholds for the two summary measures (PCS and MCS), as well as those recommended for NBS, were established some time ago and have not been frequently re-evaluated, particularly in the RA patient population. Over time, meaningful improvement scores may have changed with the improvement of treatments, more effective patient care, and increased patient awareness of disease management. A likely driver behind using the 2.5- and 5-point thresholds is the metric underlying these scores, rather than empirical findings based on analyses similar to those carried out in the current study. NBS scores are set to have a mean of 50 and a SD of 10 (based on the US general population); 0.5/0.25 of a 10-point SD is ~ 5 points and 2.5 points, which have been common metrics [24, 25].

Based on available anchors, of which SF-36 PGIS was considered the primary anchor, our analyses for RASIQ indicate a change between approximately -33 and -22 points in the JP scale score (range: 0–100) could be interpreted as being meaningful for patients; for the JS scale (range: 0–100), this range was approximately -24 to -27, while for the IM scale (range: 0–100), the range of change scores was approximately -21 to -17. For all three scales, distribution-based results indicated that the changes within these ranges were well beyond error that would occur by chance in the measurement process. Overall, anchor-based estimates were associated with high values for sensitivity, indicating that the WPMI estimates were good at identifying patients who improved; values for specificity were low, indicating that these thresholds may have included a lot of patients that were not “truly” improved. Further studies and/or assessment with other measures is therefore warranted.

### Limitations

Only anchors that were included in the two Phase 2 trials were available in the current study. These anchors were not specifically developed for the purposes of deriving WPMI thresholds and did not include patients’ direct assessment of change. As a result, for some SF-36v2 domain scales, the anchor used was not sufficiently correlated with the scale it was intended to detect signal from. RASIQ is a novel PRO instrument, hence there is limited published literature against which our findings can be compared. Due to trial assessments being too far apart, we calculated the SEM using measures of internal consistency reliability across all PROs, which is a further shortcoming given that some researchers would recommend SEM is calculated from a measure of test-test reliability. For all three PRO instruments, our analyses were limited to estimation of thresholds related to improvement. Further work is needed to estimate interpretation thresholds that indicate decline and worsening of symptoms, to confirm the values derived in the current study, and to allow exploration of the potential non-linearity across score distributions (the latter of which was not possible due to insufficient sample size).

## Conclusions

This study derived WPMI thresholds for SF-36v2, FACIT-Fatigue, and RASIQ, using multiple anchors. Derivation of WPMI thresholds for these PRO instruments will enable their broader use to assist with evaluation and interpretation of treatment benefit in future RA studies.

### Electronic supplementary material

Below is the link to the electronic supplementary material.

Additional File 1

Additional File 2

## Data Availability

The datasets used and/or analyzed during the current study are available from the corresponding author on reasonable request.

## Abbreviations

AN: anchor
BP: bodily pain
CDF: cumulative distribution function
CI: confidence interval
FACIT-Fatigue: Functional Assessment of Chronic Illness Therapy-Fatigue
GH: general health perceptions
IM: Impact
JP: Joint Pain
JS: Joint Stiffness
MCS: Mental Component Summary
MH: mental health
NBS: norm-based scores
PAIN: Patient’s Assessment of Arthritis Pain
PCS: Physical Component Summary
PF: physical functioning
PGIS: Patient’s Global Impression of Status
PRO: patient-reported outcomes
PtGA: Patient’s Global Assessment of Disease Activity
RA: rheumatoid arthritis
RASIQ: Rheumatoid Arthritis Symptoms and Impact Questionnaire
RCI: reliable change index
RE: role limitations due to emotional problems
ROC: receiver operating characteristic
RP: role limitations due to physical health
SD: standard deviation
SEM: standard error of the measurement
SF: social functioning
SF-36v2: Short-Form 36 Health Survey version 2
SRM: standardized response mean
VT: vitality
WPMI: within-patient meaningful improvement

## References

[1] Crosby RD, Kolotkin RL, Williams GR (2003). Defining clinically meaningful change in health-related quality of life. J Clin Epidemiol.

[2] Byrom B, Breedon P, Tulkki-Wilke R, Platko JV (2020). Meaningful change: defining the interpretability of changes in endpoints derived from interactive and mHealth technologies in healthcare and clinical research. J Rehabil Assist Technol Eng.

[3] US Food and Drug Administration (2019) Incorporating clinical outcome assessments into endpoints for regulatory decision-making. Available via https://www.fda.gov/media/132505/download. Accessed 19 April 2023

[4] Kalyoncu U, Dougados M, Daurès JP, Gossec L (2008). Reporting of patient-reported outcomes in recent trials in rheumatoid arthritis: a systematic literature review. Ann Rheum Dis.

[5] Becker B, Bracher M, Chauhan D, Rendas-Baum R, Lin X, Raymond K, O’Connor M, Kosinski M (2021). Development, psychometric evaluation and cognitive debriefing of the rheumatoid arthritis symptom and impact questionnaire (RASIQ). J Patient Rep Outcomes.

[6] Buckley CD, Simón-Campos JA, Zhdan V, Becker B, Davy K, Fisheleva E, Gupta A, Hawkes C, Inman D, Layton M, Mitchell N, Patel J, Saurigny D, Williamson R, Tak PP (2020). Efficacy, patient-reported outcomes, and safety of the anti-granulocyte macrophage colony-stimulating factor antibody otilimab (GSK3196165) in patients with rheumatoid arthritis: a randomised, phase 2b, dose-ranging study. Lancet Rheumatol.

[7] Genovese MC, Berkowitz M, Conaghan PG, Peterfy C, Davy K, Fisheleva E, Gupta A, Inman D, Janiczek R, Layton M, Mitchell N, Patel J, Roberts A, Saurigny D, Smith JE, Williamson R, Tak PP (2020). MRI of the joint and evaluation of the granulocyte–macrophage colony-stimulating factor–CCL17 axis in patients with rheumatoid arthritis receiving otilimab: a phase 2a randomised mechanistic study. Lancet Rheumatol.

[8] Witt S, Krauss E, Barbero MAN, Müller V, Bonniaud P, Vancheri C, Wells AU, Vasakova M, Pesci A, Klepetko W, Seeger W, Crestani B, Leidl R, Holle R, Schwarzkopf L, Guenther A (2019). Psychometric properties and minimal important differences of SF-36 in idiopathic pulmonary fibrosis. Respir Res.

[9] Gossec L, Steinberg G, Rouanet S, Combe B (2015). Fatigue in rheumatoid arthritis: quantitative findings on the efficacy of tocilizumab and on factors associated with fatigue. The French multicentre prospective PEPS study. Clin Exp Rheumatol.

[10] Jolly M, Annapureddy N, Arnaud L, Devilliers H (2019). Changes in quality of life in relation to disease activity in systemic lupus erythematosus: post-hoc analysis of the BLISS-52 trial. Lupus.

[11] Maruish ME (2011). User’s manual for the SF-36v2 Health Survey.

[12] Cella D, Yount S, Sorensen M, Chartash E, Sengupta N, Grober J (2005). Validation of the Functional Assessment of Chronic illness therapy fatigue scale relative to other instrumentation in patients with rheumatoid arthritis. J Rheumatol.

[13] Ward MM, Guthrie LC, Alba MI (2015). Clinically important changes in individual and composite measures of rheumatoid arthritis activity: thresholds applicable in clinical trials. Ann Rheum Dis.

[14] Coon C (2016). Empirical telling the interpretation story: the case for strong anchors and multiple methods. Qual Life Res.

[15] Revicki D, Hays RD, Cella D, Sloan J (2008). Recommended methods for determining responsiveness and minimally important differences for patient-reported outcomes. J Clin Epidemiol.

[16] Jacobson NS, Truax P (1991). Clinical significance: a statistical approach to defining meaningful change in psychotherapy research. J Consult Clin Psychol.

[17] Cronbach LJ (1951). Coefficient alpha and the internal structure of tests. Psychometrika.

[18] McDonald RP (1999). Test theory: a unified treatment.

[19] Sijtsma K (2009). On the use, the misuse, and the very limited usefulness of Cronbach’s alpha. Psychometrika.

[20] Norman GR, Sloan JA, Wyrwich KW (2004). The truly remarkable universality of half a standard deviation: confirmation through another look. Expert Rev Pharmacoecon Outcomes Res.

[21] van Mulligen E, Weel A, Kuijper TM, Hazes JMW, van der Helm-van Mil AHM, de Jong PHP (2020). The impact of a disease flare during tapering of DMARDs on the lives of rheumatoid arthritis patients. Semin Arthritis Rheum.

[22] Emery P, Kavanaugh A, Bao Y, Ganguli A, Mulani P (2015). Comprehensive disease control (CDC): what does achieving CDC mean for patients with rheumatoid arthritis?. Ann Rheum Dis.

[23] Bjorner JB, Terluin B, Trigg A, Hu J, Brady KJS, Griffiths P (2022). Establishing thresholds for meaningful within-individual change using longitudinal item response theory. Qual Life Res:Online Ahead of Print.

[24] Lubeck DP (2004). Patient-reported outcomes and their role iin the assessment of rheumatoid arthritis. PharmacoEconomics.

[25] Strand V, Boers M, Idzerda L, Kirwan JR, Kvien TK, Tugwell PS, Dougados M (2011). It’s good to feel better but it’s better to feel good and even better to feel good as soon as possible for as long as possible. Response criteria and the importance of change at OMERACT 10. J Rheumatol.

