# Supplementary material for: Meaningful score changes for SF-36v2, FACIT-fatigue, and RASIQ in rheumatoid arthritis
Source: J Patient Rep Outcomes. 2024 Jan 22;8:9. doi: 10.1186/s41687-024-00685-0 (PMC10806967; doi:10.1186/s41687-024-00685-0)
Supplement: Supplementary file 1 — Additional File 1 [file 41687_2024_685_MOESM1_ESM.docx]

# Additional File 1 – NBS results

*SF-36v2*

Anchor-based WPMI values based on NBS ranged between 6.5 for the GH scale and 11.7 for the VT scale. Estimates based on reliable change were generally smaller than anchor-based estimates, ranging from 4–5 points for PF, RP, BP, RE, PCS, and MCS, from 5.5–6.5 points for VT, from 6.5–7.5 points for MH, from 7–8 points for GH, and 8.5 points for the SF scale when using NBS (**Supplementary Table S1**).

**Table S1** Mean change score analysis results for SF-36v2 (NBS)

|  | **PtGA^a^** | | | | **PAIN^b^** | | | | **AN5^c^** | | | | **RCI^d^** |
| --- | --- | --- | --- | --- | --- | --- | --- | --- | --- | --- | --- | --- | --- |
|  | **N** | **Mean** | **95% CI** | **SRM** | **N** | **Mean** | **95% CI** | **SRM** | **N** | **Mean** | **95% CI** | **SRM** |  |
| **Scale/component summary (NBS)** | | | | | | | | | | | | | |
| Physical functioning | 120 | 10.3 | 8.87–11.79 | 1.27 |  |  |  |  |  |  |  |  | 5.36 |
| Role physical | 120 | 9.6 | 8.05–11.17 | 1.37 |  |  |  |  |  |  |  |  | 4.20 |
| Bodily pain | 120 | 10.7 | 9.23–12.21 | 1.65 | 125 | 11.2 | [9.83–12.65] | 1.73 |  |  |  |  | 4.68 |
| General health | 120 | 6.5 | 5.15–7.82 | 0.87 |  |  |  |  |  |  |  |  | 7.94 |
| Vitality | 120 | 10.7 | 9.10–12.39 | 1.30 |  |  |  |  | 60 | 11.7 | [9.35–13.96] | 1.41 | 6.54 |
| Social functioning | 120 | 9.0 | 7.12–10.79 | 0.98 |  |  |  |  |  |  |  |  | 8.57 |
| Role emotional | 120 | 7.9 | 6.03–9.78 | 0.70 |  |  |  |  |  |  |  |  | 5.66 |
| Mental health | 120 | 8.1 | 6.34–9.76 | 0.75 |  |  |  |  |  |  |  |  | 7.46 |
| Physical Component Summary | 120 | 9.7 | 8.33–11.12 | 1.47 |  |  |  |  |  |  |  |  | 4.05 |
| Mental Component Summary | 120 | 7.6 | 5.79–9.35 | 0.70 |  |  |  |  |  |  |  |  | 5.96 |

^a^Improvement: a decline of 18 points or more

^b^Improvement: a decline of 20 points or more

^c^Improvement: an increase of 1 point on a 5-point Likert scale

^d^RCI analyses based on Cronbach’s alpha

AN, anchor; CI, confidence interval; NBS, norm-based scoring; PAIN, Patient’s Assessment of Arthritis Pain; PtGA, Patient’s Global Assessment of Disease Activity; RCI, reliable change index; SF-36v2, Short-Form 36 Health Survey version 2; SRM, standardized response mean
