# Supplementary material for: Meaningful score changes for SF-36v2, FACIT-fatigue, and RASIQ in rheumatoid arthritis
Source: J Patient Rep Outcomes. 2024 Jan 22;8:9. doi: 10.1186/s41687-024-00685-0 (PMC10806967; doi:10.1186/s41687-024-00685-0)
Supplement: Supplementary file 2 — Additional File 2 [file 41687_2024_685_MOESM2_ESM.docx]

# Additional File 2

**Table S1** SF-36v2 ROC curve analysis

|  | | | | **Discriminant cut point** | | | | | | | | | |
| --- | --- | --- | --- | --- | --- | --- | --- | --- | --- | --- | --- | --- | --- |
| **Anchor** | **N, total** | **N, improved** | **% with improvement** | **PF** | **RP** | **BP** | **GH** | **VT** | **SF** | **RE** | **MH** | **PCS** | **MCS** |
| **NBS scores** | | | | | | | | | | | | | |
| PtGA^a^: Considering all the ways your arthritis has affected you, how active do you feel your arthritis is … | 203 | 120 | 59 | 10.52 | 7.35 | 4.65 | 3.34 | 9.37 | 10.91 | 7.77 | 5.63 | 6.99 | 5.81 |
| PAIN^b^: How much pain are you currently having because of your rheumatoid arthritis? | 203 | 125 | 62 |  |  | 8.03 |  |  |  |  |  |  |  |
| AN5^c^: I have energy. | 203 | 83 | 41 |  |  |  |  | 9.37 |  |  |  |  |  |
| **0**–**100 scores** | | | | | | | | | | | | | |
| PtGA^a^: Considering all the ways your arthritis has affected you, how active do you feel your arthritis is … | 203 | 120 | 59 | 25.00 | 18.75 | 11.00 | 7.00 | 18.75 | 25.00 | 16.67 | 10.00 |  |  |
| PAIN^b^: How much pain are you currently having because of your rheumatoid arthritis? | 203 | 125 | 62 |  |  | 19.00 |  |  |  |  |  |  |  |
| AN5^c^: I have energy. | 203 | 83 | 41 |  |  |  |  | 18.75 |  |  |  |  |  |

^a^Improvement: a decline of 18 points or more

^b^Improvement: a decline of 20 points or more

^c^Improvement: an increase of 1 point on a 5-point Likert scale

AN, anchor; BP, bodily pain; GH, general health perceptions; MCS, Mental Component Summary; MH, mental health; NBS, norm-based scores; PAIN, Patient’s Assessment of Arthritis Pain; PCS, Physical Component Summary; PF, physical functioning; PtGA, Patient’s Global Assessment of Disease Activity; RE, role limitations due to emotional problems; ROC, receiver operating characteristic; RP, role limitations due to physical health; SF, social functioning; SF-36v2, Short-Form 36 Health Survey version 2; VT, vitality

**Table S2** FACIT-Fatigue ROC curve analysis

| **Anchor** | **N, total** | **N, improved** | **% with improvement** | **Discriminant cut point** |
| --- | --- | --- | --- | --- |
| PGIS^a^: In general, would you say your health is … | 203 | 108 | 53 | 8.00 |
| PtGA^b^: Considering all the ways your arthritis has affected you,  how active do you feel your arthritis is … | 203 | 120 | 59 | 8.00 |
| VT03^a^: How much of the time during the past 4 weeks did  you feel worn out? | 203 | 109 | 54 | 9.00 |

^a^Improvement: an increase of 1 point on a 5-point Likert scale

^b^Improvement: a decline of 18 points or more

FACIT-Fatigue, Functional Assessment of Chronic Illness Therapy-Fatigue; PGIS, Patient’s Global Impression of Status; PtGA, Patient’s Global Assessment of Disease Activity; ROC, receiver operating characteristic; VT, vitality

**Figure S1** CDF plots for the SF**-**36v2 0–100 domains


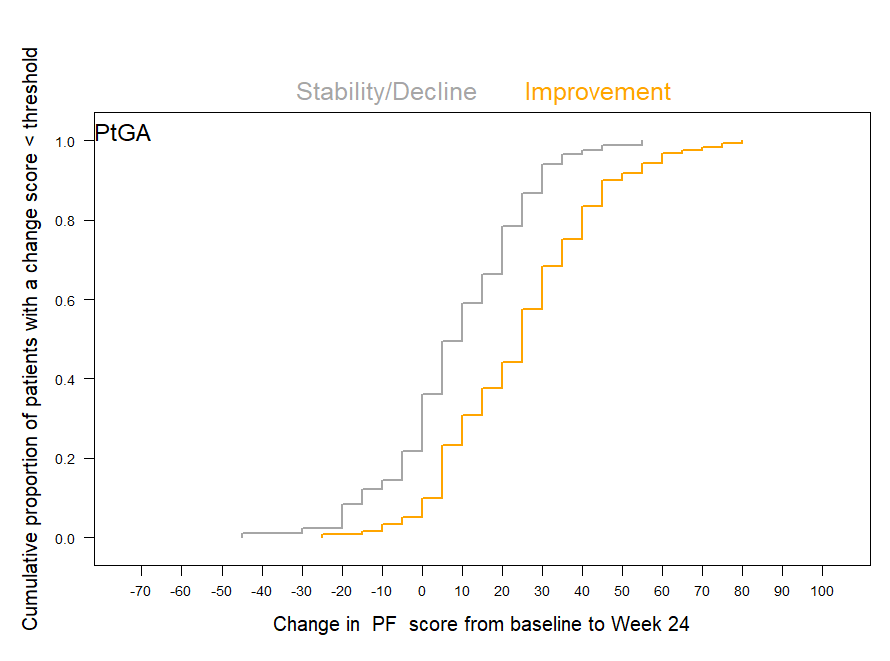

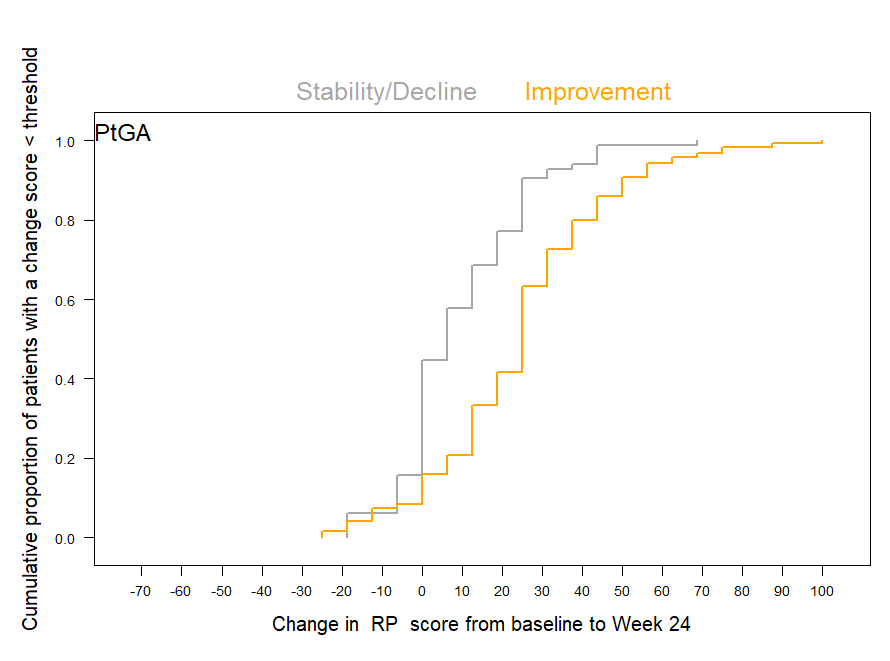


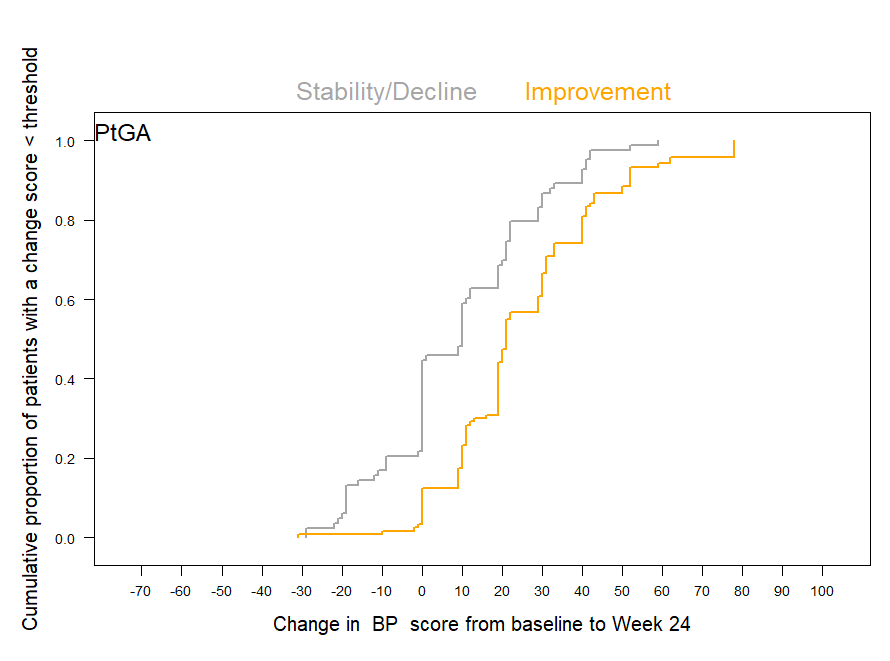


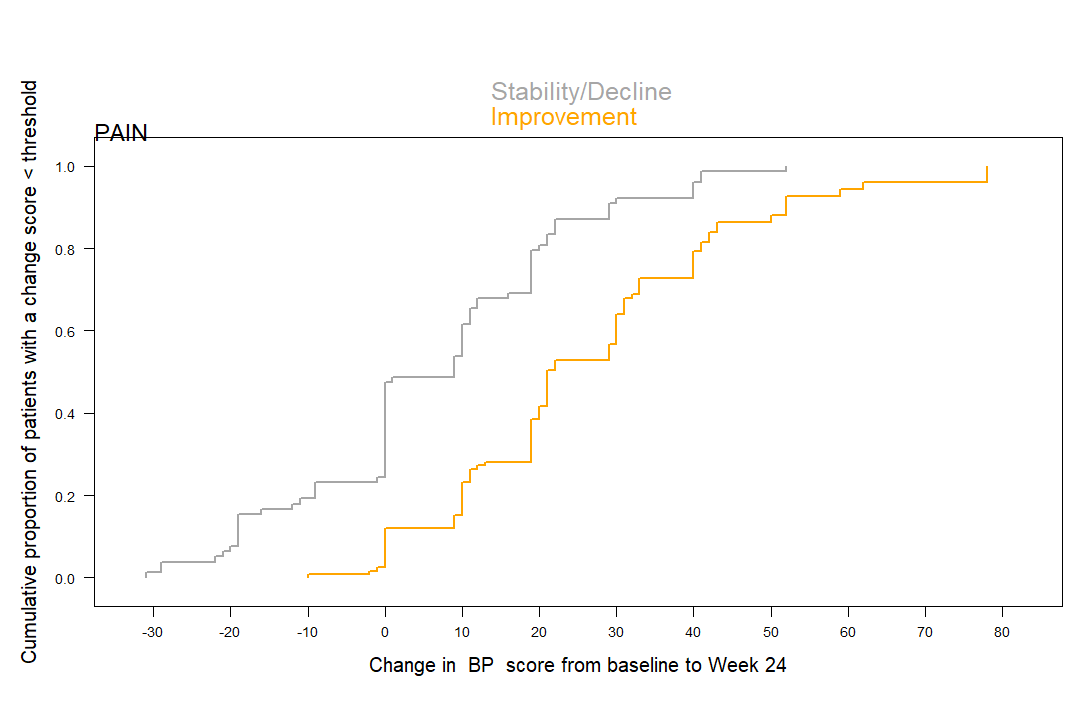


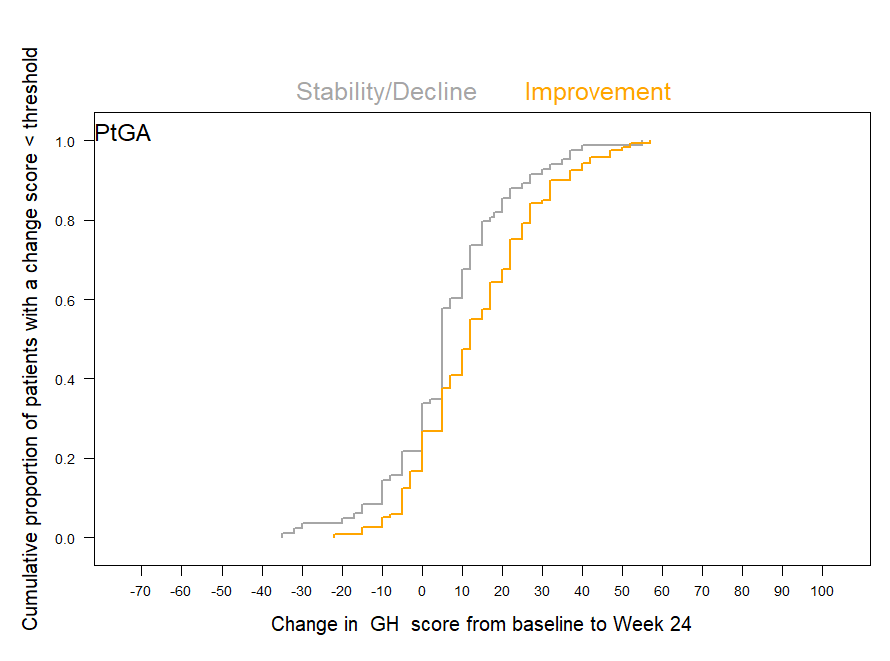


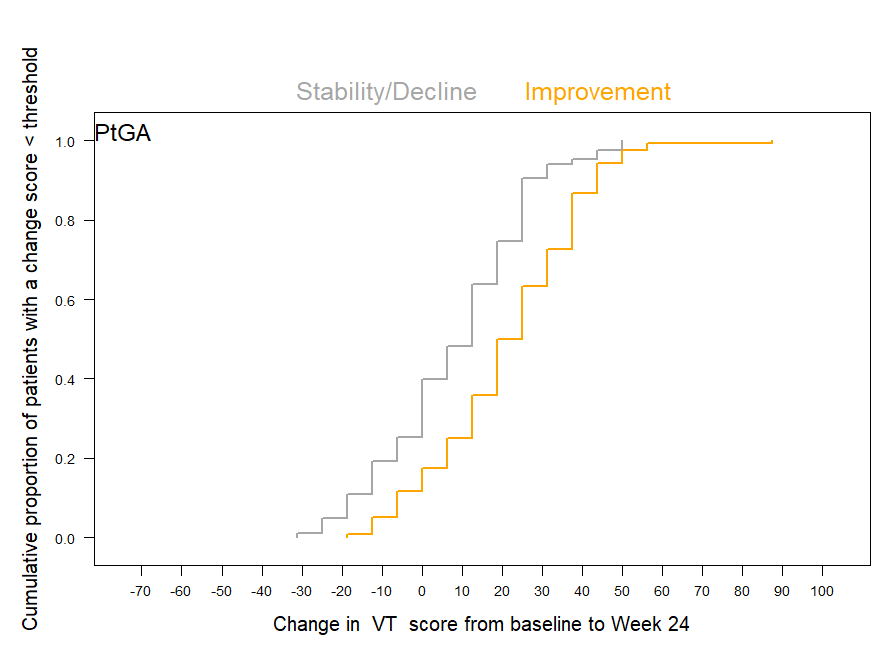


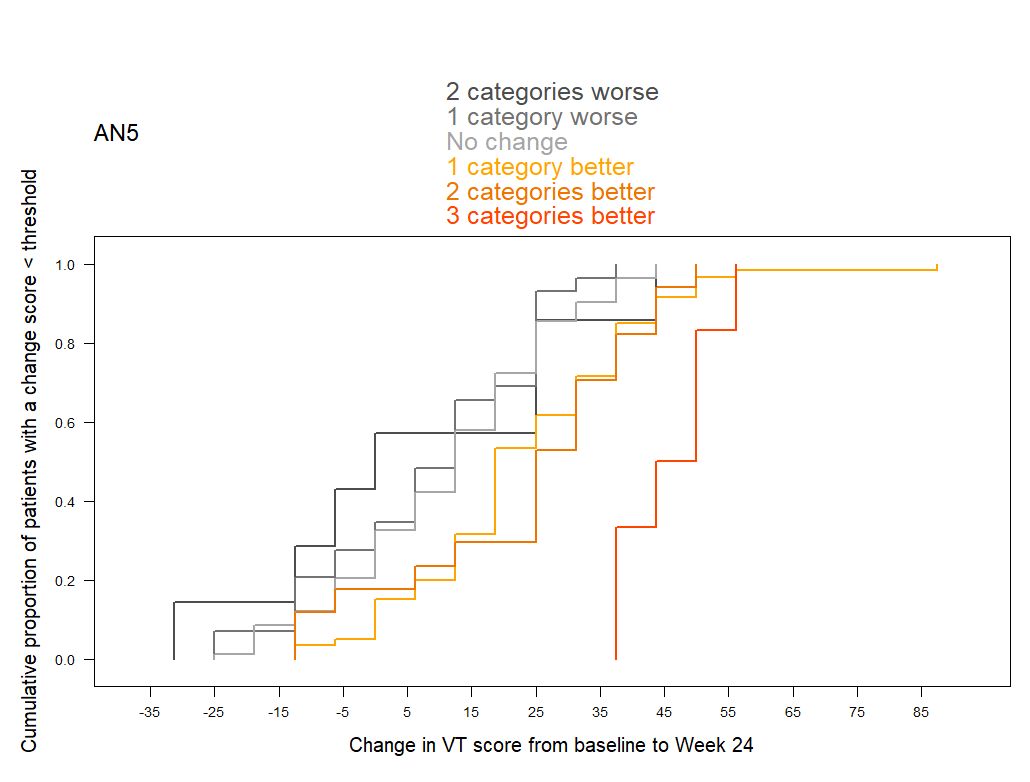


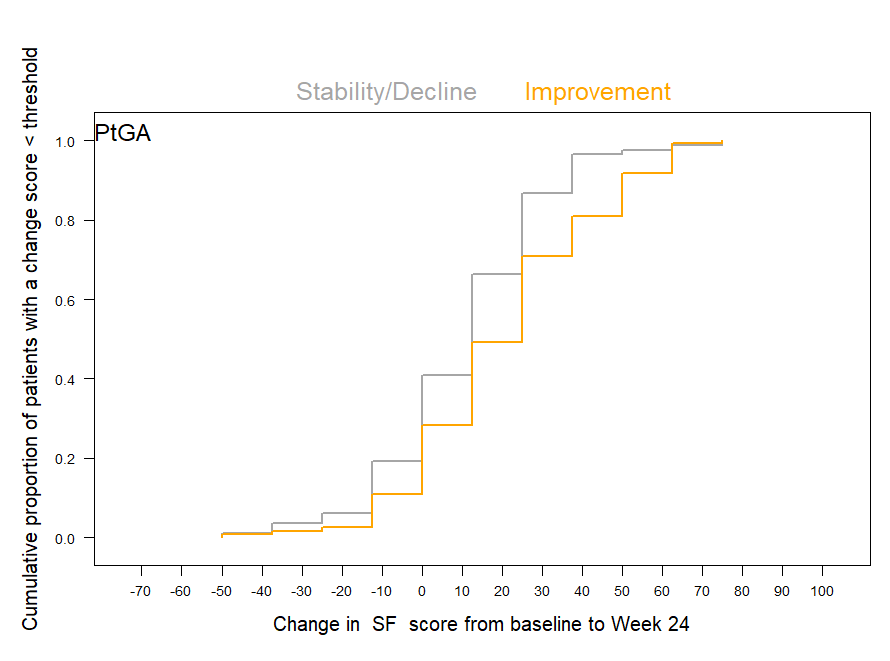


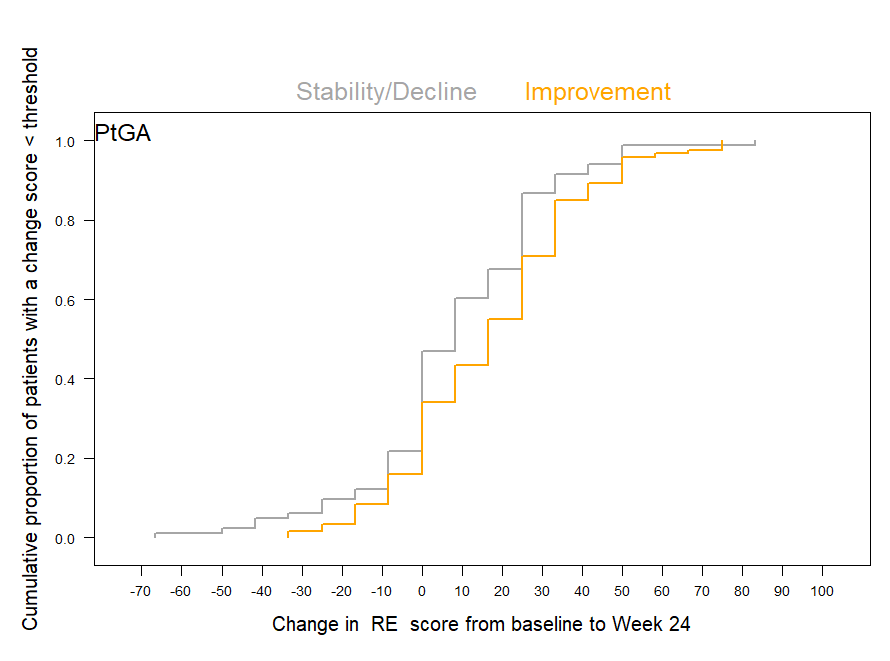


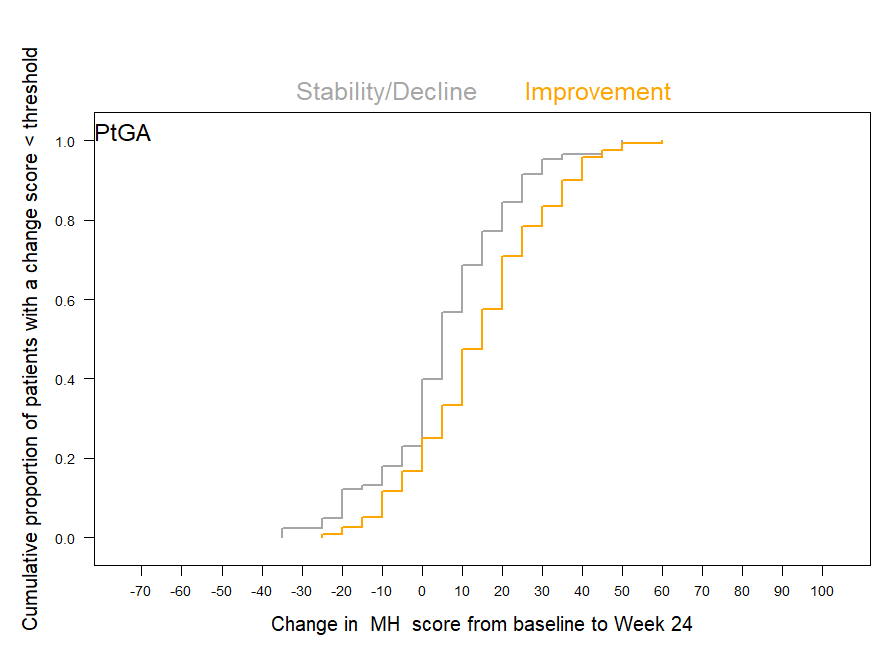


CDF, cumulative distribution function; SF-36v2, Short-Form 36 Health Survey version 2

**Figure S2** CDF plots for the SF-36v2 summary scores


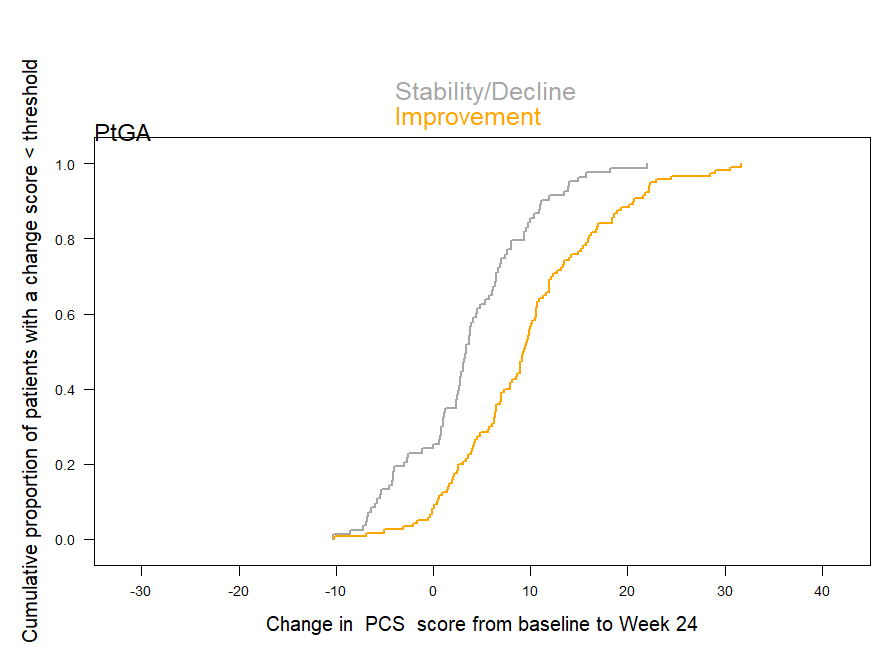


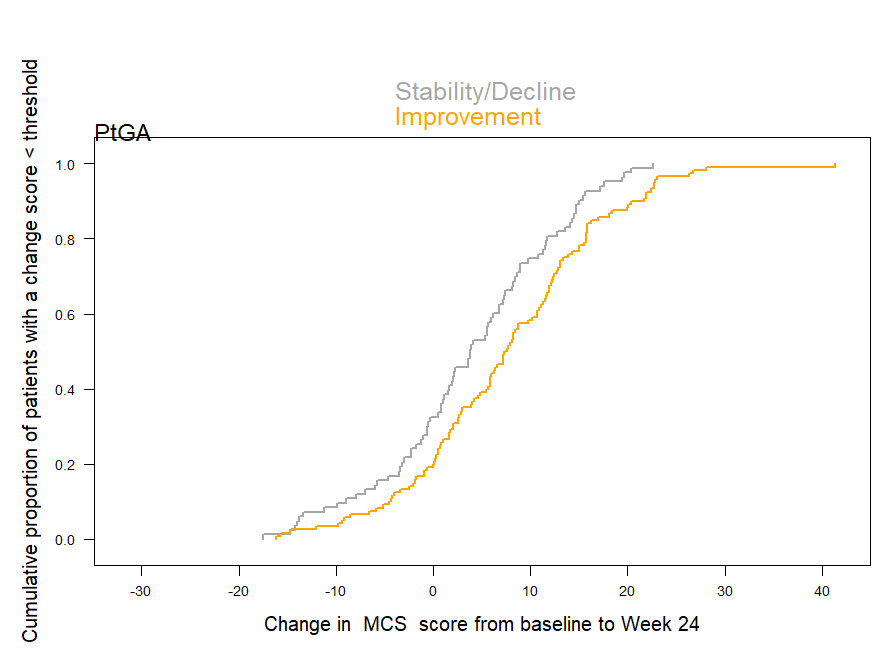


CDF, cumulative distribution function; SF-36v2, Short-Form 36 Health Survey version 2

**Figure S3** CDF plots for the FACIT-Fatigue, by anchor


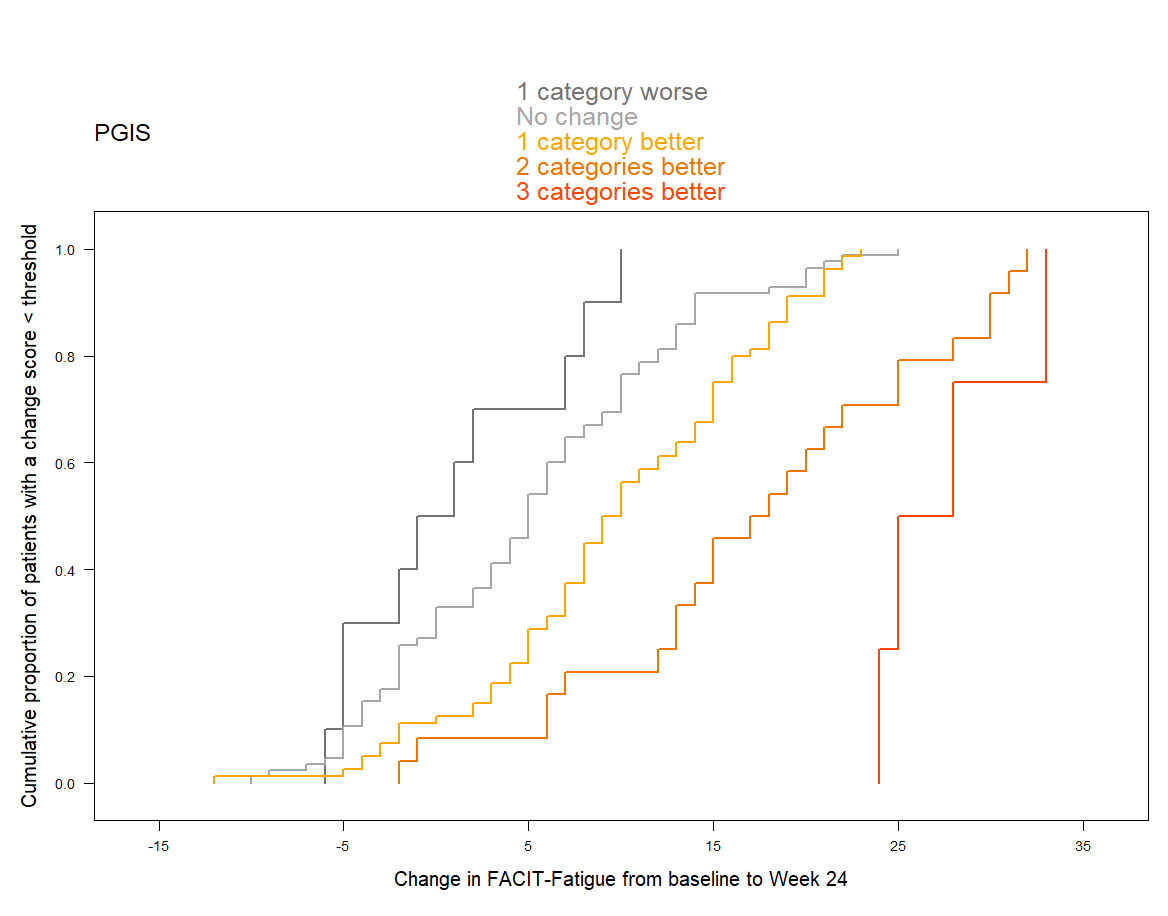


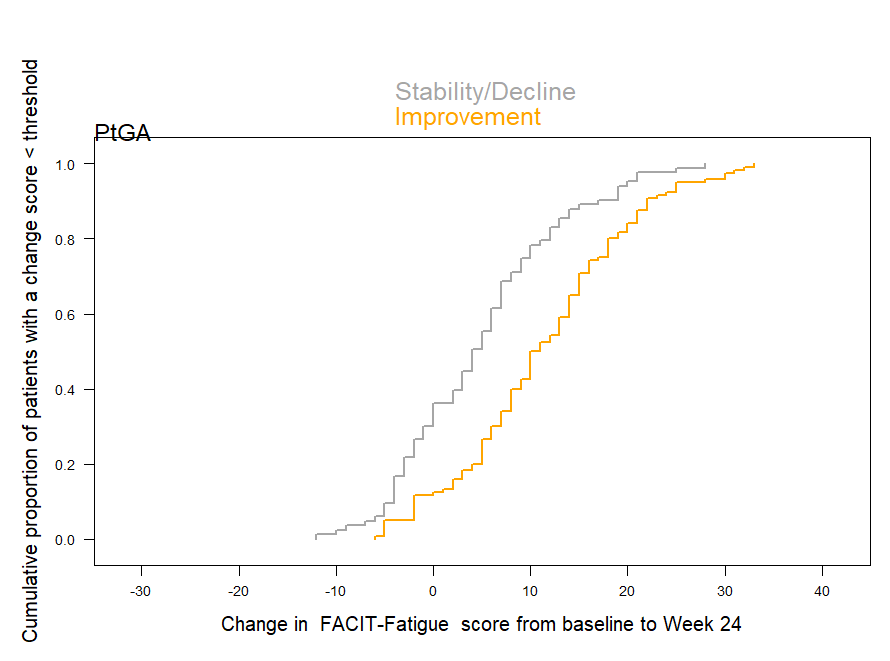


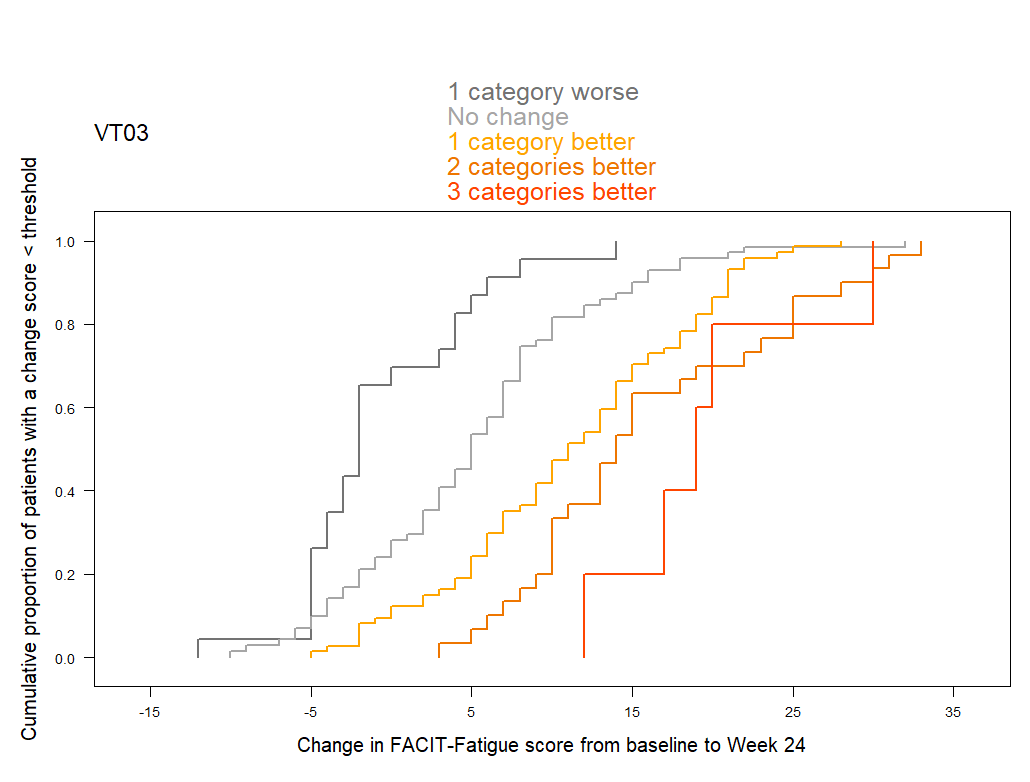


CDF, cumulative distribution function; FACIT-Fatigue, Functional Assessment of Chronic Illness Therapy-Fatigue

**Figure S4** CDF plots for the JP scale of RASIQ, by anchor


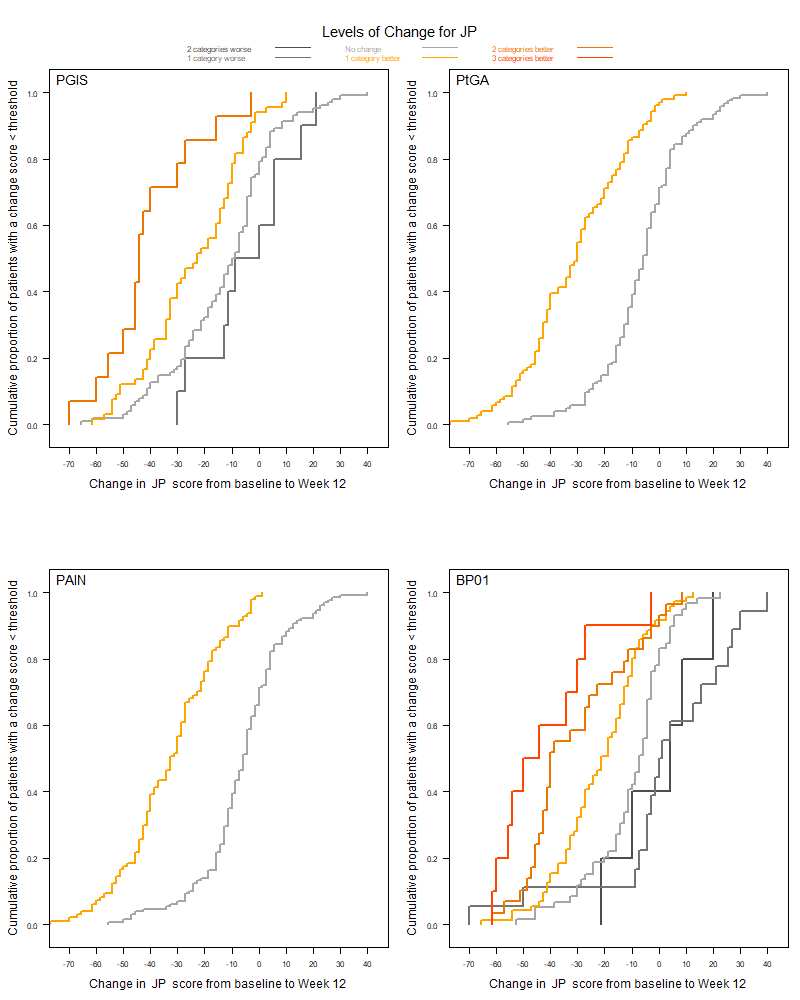


BP, bodily pain; CDF, cumulative distribution function; JP, Joint Pain; PAIN, Patient’s Assessment of Arthritis Pain; PGIS, Patient’s Global Impression of Status; PtGA, Patient’s Global Assessment of Disease Activity; RASIQ, Rheumatoid Arthritis Symptoms and Impact Questionnaire

**Figure S5** CDF plots for the JS scale of RASIQ, by anchor


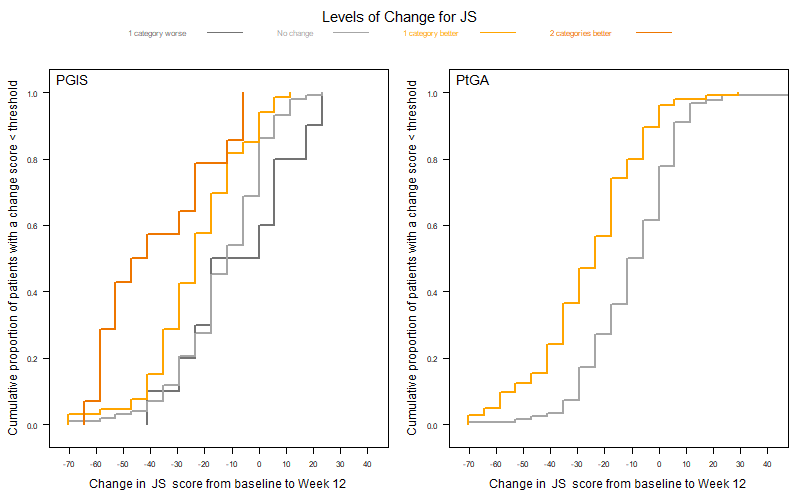


CDF, cumulative distribution function; JS, Joint Stiffness; PGIS, Patient’s Global Impression of Status; PtGA, Patient’s Global Assessment of Disease Activity; RASIQ, Rheumatoid Arthritis Symptoms and Impact Questionnaire

**Figure S6** CDF plots for the IM scale of RASIQ, by anchor


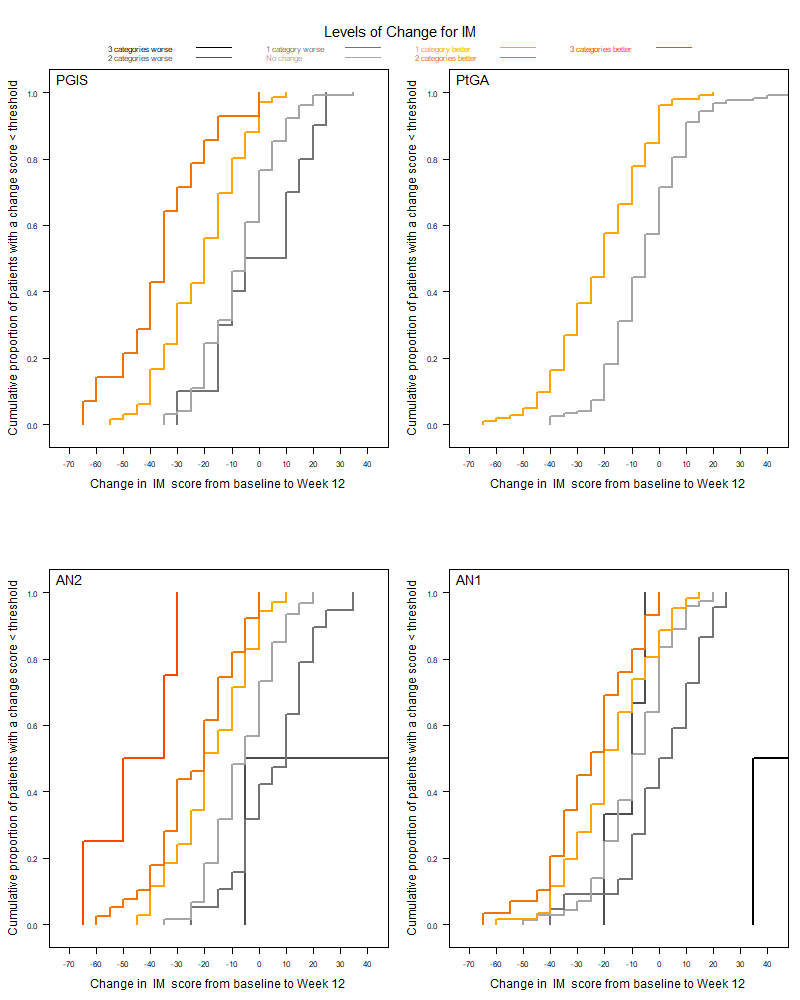


AN, anchor; CDF, cumulative distribution function; IM, Impact; PGIS, Patient’s Global Impression of Status; PtGA, Patient’s Global Assessment of Disease Activity; RASIQ, Rheumatoid Arthritis Symptoms and Impact Questionnaire
